# Zoo Visitor Attitudes Are More Influenced by Animal Behaviour than Environmental Enrichment Appearance

**DOI:** 10.3390/ani11071971

**Published:** 2021-06-30

**Authors:** Marina Salas, Daan W. Laméris, Arno Depoortere, Lise Plessers, Jonas Verspeek

**Affiliations:** 1 Antwerp Zoo Centre for Research and Conservation (CRC), Royal Zoological Society of Antwerp (KMDA), 2018 Antwerp, Belgium; daan.lameris@kmda.org (D.W.L.); Jonas.Verspeek@kmda.org (J.V.); 2 Behavioural Ecology and Ecophysiology Group, Department of Biology, University of Antwerp, 2020 Antwerp, Belgium; 3 Faculty of Psychology and Educational Sciences, KU Leuven, 3000 Leuven, Belgium; arno.depoortere@kuleuven.be (A.D.); liseplessers@hotmail.com (L.P.)

**Keywords:** animal–visitor interactions, behaviour, captivity, enrichment, visitor, welfare, zoo-housed

## Abstract

**Simple Summary:**

Environmental enrichment is a combination of techniques that aim to improve the quality of life of zoo animals. However, institutions might be reluctant to add certain enrichment items due to the belief that their presence could negatively affect the visitor experience in the zoo. To explore the veracity of this belief, we assessed visitor attitudes towards two types of enrichment items (naturalistic vs. artificial looking) in an outdoor walk-through enclosure for ring-tailed lemurs in Zoo Planckendael (Belgium). We developed a questionnaire that was answered by 371 visitors. We also took into consideration the behaviour of the animals and their visibility. We found that the visitor attitudes were more influenced by the behaviours displayed by the lemurs than the appearance of the enrichment items. We suggest that more emphasis should be placed on designing enrichment items that provide the animals with opportunities to display more active and appropriate behaviours, regardless of the appearance of the objects, in order to improve animal welfare while simultaneously improving the visitor experience.

**Abstract:**

Decisions on environmental enrichment programmes are sometimes based on the assumption that non-natural or artificial looking items negatively affect visitor experiences. In this study, we developed a questionnaire to assess zoo visitor attitudes towards enrichment appearance in an outdoor walk-through enclosure for ring-tailed lemurs (*Lemur catta*). Naturalistic and artificial looking enrichment items were alternately provided in the enclosure. A total of 371 visitors filled out the questionnaire: 174 in the naturalistic and 197 in the artificial conditions. Both researchers and visitors conducted behavioural observations of the lemurs. Our results suggest that the appearance of the items did not have an effect on visitor attitudes and that visitors recognised both naturalistic and artificial items as enriching for the animals. Moreover, the behaviour and visibility of the lemurs had a greater effect on the visitors’ attitudes. We suggest that during the design of enrichment items, less concern should be placed on the appearance of the items and more on their effect on animal behaviour. Ultimately, this would improve both animal welfare in captivity and the visitor experience.

## 1. Introduction

It is generally accepted that modern zoos have four roles, including conservation, education, research, and entertainment of the visitors [1]. The role of animal–visitor interactions (AVI) is often more focused on the impact such AVIs have on the welfare of animals, and less on zoo visitors. Yet, previous studies suggest that AVIs influence zoo visitor experiences. Spooner et al. examined the effects on the visitor experience of both animal shows [2] and educational theatre [3] at zoos. They found that live animal shows but also family theatre without live animals were effective in increasing visitors’ knowledge on basic animal features as well as awareness of the zoos’ conservation efforts. Additionally, public animal-training sessions produced more positive zoo experiences for visitors [4].

The effects of zoo animal behaviour on the perceptions and behaviours of visitors have also been studied on several occasions. For instance, Altman [5] observed that the more ‘animated activities’ displayed by polar bears (*Ursus arctos maritimus*), the more the visitor attention was directed to the animals and their behaviours, as showed by an increase in the behaviour content in the conversations among the visitors. Godinez et al. [6] and Miller [7] examined visitor responses to the behavioural displays of zoo animals, with particular attention to stereotypies. Negative visitor perceptions were related to the animals being out of sight [6] or displaying stereotypic behaviours [6,7]. Tofield et al. [8] examined both naturalistic exhibits and behavioural displays on zoo visitor reports taken from interviews and observed that visitors found enriched exhibits more attractive and spent more time at more naturalistic enclosures.

Environmental enrichment is a combination of techniques with the aim to enhance the quality of life of zoo-housed animals by providing environmental stimuli necessary for optimal psychological and physiological wellbeing [9,10,11,12]. Good environmental enrichment programmes facilitate the expression of species-specific behaviours by increasing behavioural diversity [9,10,13,14], including extending foraging time and problem-solving [15], reducing extended periods of inactivity and undesired behaviours [15,16,17,18] and lowering the frequency of aggressive behaviours [13,19]. Environmental enrichment programmes also aim to increase the animal’s sense of control and ability to choose [12], which is an important contributor to animal welfare since the perception of unpredictable situations, being unable to choose and not having a sense of control over the environment, have been related with stress [11].

Environmental enrichment, as noted by Mellen and MacPhee [9], is a holistic approach that involves both the enclosures where animals are housed and the stimuli and events that occur within such enclosures. Over the last few decades, animal enclosures have transitioned to naturalistic environments to enhance visitor attitudes and improve animal welfare [1,9,20,21,22,23]. The environment surrounding an animal influences the visitor perception of that animal [24,25] and visitors show a preference for more naturalistic enclosures [24]. Naturalistic enclosures tend to be more complex and, therefore, improve animal welfare. In addition to the permanent elements of the enclosure design, often more temporary environmental enrichment objects are added. These objects can either be naturalistic or artificial looking, mostly depending on the materials used to build them. The design and provision of environmental enrichment relies heavily on the enthusiasm and dedication of motivated animal caretakers. However, in order to succeed, enrichment programmes need support from other entities within the zoo, such as directors, curators, managers, veterinary and other zoo staff [9,26]. Still, institutions may be reluctant to add certain enrichment devices due to the (untested) assumption that their presence can negatively affect the visitor experience, especially in a more naturalistic enclosure [21,27]. One way to overcome this problem, is to provide naturalistic looking items. Indeed, it has been shown that naturalistic exhibit designs are more attractive and informative to the visitors [1,20,21,24,25] and it could be argued that natural looking enrichment would add to the cohesion of a naturalistic enclosure [28,29]. However, artificial materials are often considered cheaper, more durable, easier to be cleaned and disinfected, as well as easier to modify without compromising the structure of the material, compared to more natural looking materials [30]. This could be a reason why environmental enrichment (especially artificial looking items) is sometimes provided off-exhibit, in areas not accessible to public view [21]. Yet, it has been suggested that animals in an enriched environment are more likely to exhibit active and species-appropriate behaviour and this is probably more informative and interesting to zoo visitors [9,22,23,26]. Visitor attitudes are of importance to the zoo management and a positive influence on visitor experience would thus be an additional benefit of enrichment. Davey et al. [14] assessed visitor behavioural responses to environmental enrichment improvements in a mandrill (*Mandrillus sphinx*) enclosure at Beijing zoo (China). They observed that the improved enclosure attracted more visitors, who stayed longer, suggesting that the enriched design was more attractive than the previous exhibit. However, they did not compare artificial with naturalistic looking enrichment, but a barren enclosure with a remodelled naturalistic exhibit with enrichment devices.

Most studies on the impact of environmental enrichment on zoo visitors’ experience suggested that a mismatch between enrichment appearance and exhibit design has little effect on the attitudes of the visitor [21,27,29,31]. Jacobson et al. [31] explored the effect of artificial looking enrichment on visitor perceptions in a naturalistic chimpanzee (*Pan troglodytes*) enclosure in Lincoln Park Zoo (USA). They concluded that the visitors were not affected by the aesthetics of the enrichment and showed that the largest proportion of visitors thought the naturalism of the enclosure was important. Similar results are reported in a study investigating the effects of enrichment in a polar bear enclosure on visitor perception: enrichment type, either artificial or naturalistic looking, did not alter visitor perceptions about the zoo, the enclosure, the species on display, or the individual animals [21]. McPhee et al. [27] surveyed zoo visitors about their perceptions of naturalism in polar bear, tiger (*Panthera tigris altaica*), lynx (*Lynx canadensis*), and fishing cat (*Prionailurus viverrinus*) exhibits in Brookfield Zoo (USA), but their results did not report an influence of the type of enrichment on visitor impressions.

In another study conducted at Brookfield Zoo [28], visitors were asked to score videos and photographs of different exhibits, with or without animals, and with different types of enrichment, naturalistic or artificial looking. Contrary to previous research, Razal and Miller’s results suggested that different types of enrichment, as well as the naturalistic appearance of the enclosure, might have an impact on how visitors perceive animals and their exhibits. They found that visitors rated the pictures with naturalistic looking enrichment higher for questions related with good animal welfare and the enclosure suitability and naturalness. Visitors also ranked the naturalistic looking enrichment items higher.

According to Luebke and Matiasek [32], the most common factors that impact visitors’ experience in zoos involve not only exhibit features, but also animal visibility and behavioural activity. Most of the studies on the impact of environmental enrichment in visitors, though, did not control for animal visibility and behaviour. To better comprehend visitor perception of enrichment, it is necessary to have more studies that control for animal behaviour and visibility [28].

Since cultural background has been shown to have an effect on attitudes towards animals and animal welfare [33], visitor experience regarding the presence of environmental enrichment might also differ between human societies. To our knowledge, all the studies that assessed visitor attitudes towards enrichment or how enrichment influences visitors’ experiences have been conducted in the United Kingdom [29], the United States of America [21,27,28,31] or China [14].

The aim of this study was to assess visitor attitudes towards enrichment appearance on a walk-through enclosure for ring-tailed lemurs (*Lemur catta*) in an European zoo. Our goal was also to determine if the visibility and behaviour of the lemurs made a difference on visitor attitudes towards the enrichment devices. We expected that visitor attitudes did not differ between naturalistic and artificial looking enrichment [21,27,31], but that lemur behaviour had an influence instead [32].

## 2. Materials and Methods

### 2.1. Study Subjects

This study was conducted in the ring-tailed lemur enclosure in Zoo Planckendael (Belgium) for 10 days in April 2019. The enclosure consisted of a walk-through outdoor enclosure, a visible indoor enclosure (but inaccessible to the animals at the time of the study) and an off-exhibit indoor enclosure, which was not visible from the visitor side. The outdoor enclosure had a natural looking design that included vegetation, grass, climbing structures (including trees), and a pond. Visitors were allowed to walk on a designated path through the outdoor enclosure, where six ring-tailed lemurs roamed (Table 1). It was forbidden for visitors to touch or feed the lemurs. The outdoor enclosure was 545 m^2^ and the indoor enclosure was 48 m^2^.

In total, 371 zoo visitors participated in the study; 174 visitors participated on days with naturalistic looking enrichment and 197 visitors participated on days with artificial looking enrichment. The information related with the visitors that were interviewed can be found in Table 2.

### 2.2. Visitor Attitudes Questionnaire

To collect visitor attitudes, a questionnaire was developed, consisting of two parts. The first part included 14 attitude statements and one multiple-choice question on the behaviour of the lemurs. The participant had to indicate for each of the 14 statements on a 7-point Likert scale to what degree they agreed with the statement, ranging from ‘Completely disagree’ (score = 1) to ‘Completely agree’ (score = 7), with a neutral midpoint at score 4. In the multiple-choice question on lemur behaviour, visitors were asked to report whether they had seen any social behaviours, feeding behaviours, inactivity and/or activity. This question was included to collect data on the visitor perception on the lemur behaviour. The second part of the questionnaire focused on environmental enrichment and included a short text explaining the purpose of enrichment and five supporting images of examples of enrichment items in other species. After reading this text, participants were asked to score three enrichment-related statements using the 7-point Likert scale. In this section, the open-ended question ‘Do you see any enrichment items in this enclosure?’ was also included to ensure that researchers and visitors were considering the same objects as enrichment items.

To avoid order-effects [34], three versions of the questionnaire were made by varying the order of the statements. For each participant, the starting and ending times were collected to determine the questionnaire interval. The questionnaire was originally in Dutch and it was randomly offered to adult visitors on a clipboard while they were walking through the enclosure. They were asked by the researcher to read and complete on their own a questionnaire on housing of the ring-tailed lemurs. Visitors were in no way informed about the appearance of the enrichment items, nor was their attention directed to the enrichment items by the researchers. Evaluation of the enrichment items was only requested halfway into the questionnaire, when the researcher had already left the visitors to complete the questionnaire on their own. To avoid interviewer and response biases, visitors were assured in the introductory text that their answers would be anonymous [34].

### 2.3. Enrichment Items

**Figure 1:**
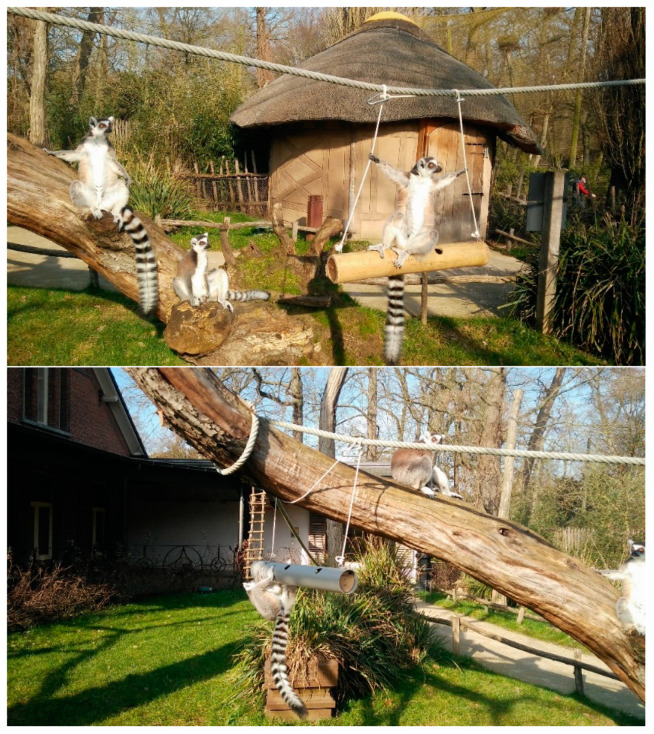
Ring-tailed lemurs interacting with environmental enrichment items: naturalistic looking (**top**) and artificial looking (**bottom**) tube swings.

Five enrichment items were designed, taking into account the ring-tailed lemurs’ needs and the behaviours that we wanted the animals to display when using them. The designs were approved for safety by the zoo veterinarian, the curator and the caretakers’ coordinator. We built two versions of each item: one naturalistic and one artificial looking (Figure 1). Among the materials the zoo directive considered natural looking were wood, rope, burlap, and beige fire hose. Plastic, PVC, bright colours, and steal were considered artificial-looking material by the zoo directive and they were what we used for the design. A description of the items can be found in Table 3.

Any time a food-related item was provided to the lemurs, two or more of the same devices were present to avoid feeding competition [10,17,19]. In these items, a third of the lemurs’ daily diet was included to avoid overfeeding. All devices used in this study had been previously offered to the lemurs during another research conducted by our group [35], and were therefore not novel to the animals.

### 2.4. Experimental Conditions

In order to determine the effect of enrichment appearance, we designed two experimental conditions that consisted of the presence of either naturalistic looking or artificial looking enrichment items in the ring-tailed lemur outdoor enclosure. To avoid habituation to the enrichment items [17,22], three out of five enrichment items were presented each day, varying between combinations of three items, over the 10 days of data collection. The enrichment items on display were always either all naturalistic looking or all artificial looking, never were the two types of enrichment displayed simultaneously. The specific combinations of enrichment items were determined pseudo-randomly beforehand, assuring that all items got an equal amount of time to be displayed.

### 2.5. Ring-Tailed Lemur Behavioural Observations

While the visitors were completing the questionnaire, we observed the behaviour of the ring-tailed lemurs. We used a focal continuous sampling method [5] and a simple ethogram (in Table 4). As we had the time when each visitor started and ended filling the questionnaire, the activity data could afterwards be linked to the individual visitor attitudes. Observational data were collected using ZooMonitor [36].

### 2.6. Statistical Analysis

First, to assess if visitor attitudes differed between conditions (having seen naturalistic or artificial looking items), a Mann–Whitney U test was performed on the 17 attitude statements. Shapiro–Wilk tests (using the build-in R function *shapiro.test*) showed the Likert-scale data to be non-normally distributed (*p*-values < 0.05). As the normality assumption for performing t-test was not met, alternatively nonparametric Mann–Whitney U tests were run to compare scores of agreement on each of the statements between the two experimental conditions (naturalistic vs. artificial looking enrichment). As a means for correction for multiple testing, we recalculated p-values by the Holm–Bonferroni method (using the build-in R function *p.adjust*).

Second, in order to investigate the relationship between visitor attitudes and lemur behaviour, we linked the questionnaire answers of each visitor to the behaviours of the lemurs during the same questionnaire interval. Two ordinal logistic regression models were performed to assess whether lemur activity level and behaviour influenced visitor attitudes. The first one was based on the data reported by the multiple-choice question by the visitors, and the second one was based on the behavioural observations carried out by the researchers. The multiple-choice question asked the visitors what the lemurs were doing during the time of their visit. They could choose one or more of the following options: (a) Feeding, drinking, foraging; (b) Inactive (sitting, resting, sleeping); (c) Active (walking, running, jumping, climbing; (d) Social behaviour (playing, grooming); and/or (e) Other. For this question, we did not want to draw the visitor’s attention towards the enrichment items, so we did not include ‘Interaction with enrichment’ as a behaviour in the list. A researcher observed the lemur behaviour during the time window where each participant was taking the survey (questionnaire interval). For each lemur, it was observed if they were active, inactive, displaying social behaviours, or out of view (if they were indoors), and if they were using an enrichment device or not. The total number of seconds that the lemurs exhibited behaviour of a certain category—sum of all lemurs—was divided by the total sum of all lemur behaviours (in seconds) exhibited during the questionnaire interval to get the proportion of each behavioural category for each participant. Then, we analysed the relation between the proportions of lemur behaviour of each category and the scores of the participants for each of the Likert-type questions.

Finally, for the open question ‘Do you see any enrichment items in this enclosure?’, percentages were calculated for four pre-defined response categories. These categories included: (1) Enrichment items designed for this study and swinging rings permanently present on the enclosure; (2) Other items or features of the enclosure; (3) Sounds and musical background; (4) People (including visitors and caretakers).

Mann–Whitney U tests, explorative analyses, linking researcher observed lemur activity to participant questionnaires and ordinal logistic regressions (for what the rms package [37] was used) were performed in R studio (R Development Core Team, 2016).

### 2.7. Ethical Statement

The Royal Zoological Society of Antwerp waived the requirement for formal ethical approval of this study for the following reasons, regarding animal welfare and visitor participation. Concerning animal welfare, this study was conducted in compliance with relevant Belgian and European legislation, and in agreement with international and scientific standards and guidelines. Due to the non-invasive character of the study, and absence of any potential discomfort, our study does not meet the definition of an animal experiment as mentioned in Chapter I, Article 16 of the Belgian ‘Act on the protection and wellbeing of animals’ (Wet van 14 augustus 1986 betreffende de bescherming en het welzijn der dieren gesynchroniseerd met de wet van 27 December 2012). Regarding visitor participation, trained zoo staff and research interns approached adult visitors in the outdoor walk-through exhibit for the ring-tailed lemurs and asked them if they were willing to participate voluntarily in the survey. All subjects who agreed to participate gave their verbal informed consent for inclusion in this study before completing the printed questionnaire. No personal information was collected, and all participants were assured in the introductory text that participation in the questionnaire would be completely anonymous. This study did not require ethical approval since it did not involve any interventions or handling of the animals. The enrichment items used in this study were evaluated and approved by the veterinarian, curator, and caregiver coordinator of Zoo Planckendael.

## 3. Results

### 3.1. Visitor Attitudes towards Naturalistic and Artificial Looking Enrichment

**Figure 2:**
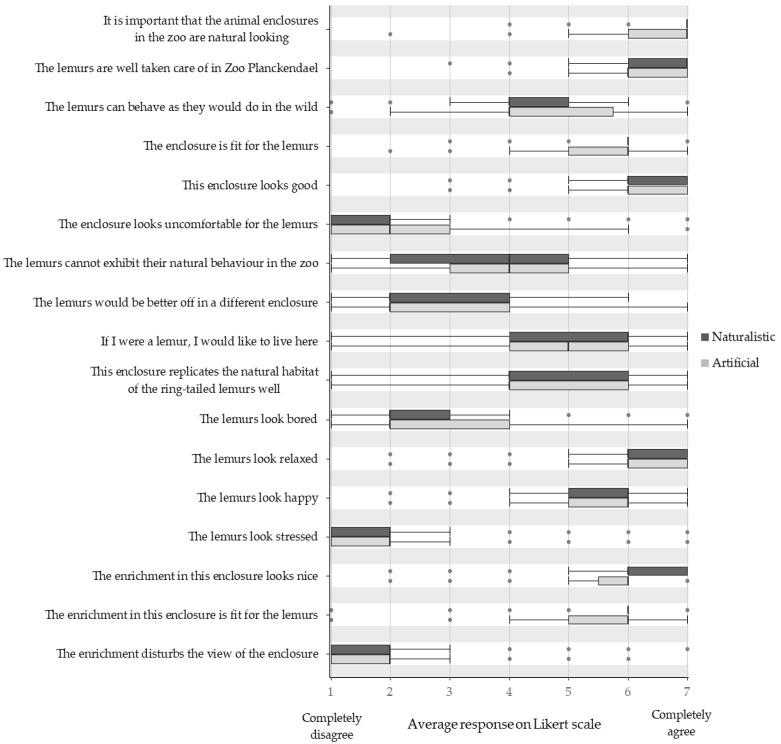
Comparison of the average scores given by the visitors when observing naturalistic and artificial looking enrichment items. All scores were given on a 7-point Likert scale ranging from ‘1 = Completely disagree’ to ‘7 = Completely agree’ with a neutral midpoint at score 4.

Visitor attitude scores were compared between the naturalistic and the artificial condition for all 17 questionnaire statements (Figure 2). No statistically significant differences were found in any of the statements.

### 3.2. Relationship between Visitor Attitudes and the Lemur Behaviours as Observed by the Researchers

Ordinal logistic regression analyses were conducted to investigate the relation between lemur behaviour—the proportions of time the lemurs were active, inactive, inside (out of view for the participants) and engaging with the enrichment items during the time window where the participant was completing the survey—and participants’ attitude scores on each of the survey questions.

When the lemurs showed more active behaviour, visitors tended to agree more with the following statements: *‘I think the enrichment in this enclosure is fit for the lemurs’* and ‘*The lemurs look happy*’. The time that the lemurs spent interacting with the enrichment was significantly related to higher Likert-scores on the following statement: ‘*I think the enrichment in this enclosure is fit for the lemurs*’ (Table 5).

### 3.3. Relationship between Visitor Attitudes and the Lemur Behaviours as Perceived by the Visitors

Concerning the results on the behaviours the visitors reported in the multiple-choice question (Table 6), we found that when visitors saw social behaviour (binary: yes or no), they provided significantly lower Likert-scores on the statements ‘*The lemurs look stressed*’ and *‘I think the enrichment disturbs the view of the enclosure’*, and higher scores on the statements: ‘*I think the lemurs can behave as they would do in the wild*’, ‘*I think this enclosure replicates the natural habitat of the ring-tailed lemurs well*’, ‘*The lemurs look relaxed*’ and ‘*The lemurs look happy*’.

When visitors observed the lemurs during feeding, they agreed significantly more with the statement ‘*I think the enrichment in this enclosure is fit for the lemurs*’ and less with the statement ‘*I think the enrichment disturbs the view of the enclosure*’.

If visitors reported seeing inactivity of the lemurs during their visit, they agreed significantly more with ‘*The lemurs look bored*’.

### 3.4. Results to the Open Question

The analysis of the answers to the open question ‘Do you see any enrichment items in this enclosure?’ showed that out of the 174 visitors that saw naturalistic-looking items, 89% mentioned one of our enrichment items, and 58% mentioned other features of the enclosure. Out of the 197 visitors who saw artificial looking items, 77% mentioned one of our items, and 52% mentioned other features of the enclosure. In the naturalistic-looking condition, none of the visitors considered sounds enriching, and 0.6% (1 visitor) mentioned people as enriching. In the artificial looking condition, 1% (2 visitors) considered sounds and the presence of people enriching for the lemurs.

## 4. Discussion

The aims of our study were to determine if visitor attitudes were influenced by the appearance of enrichment items and to explore whether the perceived activity levels and the observed activity levels of ring-tailed lemurs had an effect on visitor attitudes towards these items. The results obtained from the open-ended question ensured that researchers and visitors considered the same objects as enrichment items. Above three quarters of the visitors in each condition (89% in the naturalistic looking condition and 77% in the artificial) mentioned at least one of our items as enriching.

The analysis of the attitude statements of the 371 completed questionnaires showed that statements regarding the behaviour of the animals and their natural habitat elicited more neutral responses from the visitors, indicating that the visitors did not have a strong opinion about these aspects. As for the other statements, visitors responded generally in favour of the zoo, its management and care: lemurs appeared well taken care of, relaxed and happy, not stressed, and their exhibit and the enrichment in it were rated good-looking.

Our results showed that enrichment appearance did not have an influence on visitor attitudes. Visitors generally reacted positively to all enrichment items, naturalistic and artificial alike, and recognised the purpose of both naturalistic and artificial items as being enriching for the animals. The fact that the lemur enclosure in Zoo Planckendael is an immersive experience, where visitors walk through the enclosure following a pathway, might make the visitors more flexible to accept artificial enrichment devices, compared with naturalistic enclosures that lack such constructed elements. Nevertheless, these results are in line with those already published on visitor attitudes towards enrichment performed in other, non-European, zoos [21,27,31].

Providing appropriate enrichment items can increase the activity level and natural behaviour of the animals by offering them opportunities to engage in species-appropriate behaviours [19,35,38]. It has been suggested that observing active and engaged animals could promote positive visitor perceptions towards them [28]. Indeed, in our study, we observed that lemur activity levels and behaviour influenced visitor attitudes, more than the appearance of the enrichment items. Visitors scored higher in positive attitudes when they saw the lemurs displaying more active behaviours and interacting with the enrichment devices. In general, when lemurs were active, visitors increasingly reported they looked happy and that the enrichment in the enclosure was fit for the lemurs. When lemurs were inactive, meaning they were asleep or resting, visitors increasingly reported they looked bored. Moreover, when the visitors reported the lemurs displaying social behaviours like grooming or playing together, visitors considered that the animals were behaving as they would in the wild, that their enclosure mimicked their wild environment well, and that the enrichment items did not disturb the view of the enclosure. Visitors further believed that, when they saw social behaviours, the lemurs looked relaxed, happy, and not stressed both with naturalistic and artificial looking enrichment.

Importantly, when lemurs were interacting with the enrichment items, visitors provided higher scores for enclosure suitability. Moreover, when seeing the lemurs feeding on a food item from a food-based enrichment device, visitors found the enrichment items to disturb the view of the enclosure less and agreed more on that the enrichment was suited for lemurs.

The more active behaviours were displayed by the lemurs as observed by the researchers, the more positive visitor attitudes about the enclosure, the enrichment, and the lemurs’ emotional state. We argue that the functionality, rather than the appearance, of the enrichment items is important, considering its efficiency to elicit the display of more active and species-specific natural behaviours [31], which in turn enhances visitors’ zoo experience. The study of enrichment functionality was not directly addressed by the current study, but preference assessments have been used to test preferred food items in enrichment devices in four species of lemurs [39]. The effects of visitors on the behaviours of ring-tailed [40] and crowned (*Eulemur coronatus*) [41] lemurs in walk-through exhibits have also been examined. Further research could include the study of the relationship between type of enrichment, lemur behaviour, and the perceptions, attitudes and behaviours of the visitors. Moreover, what similar exhibits or factors related to enrichment within such exhibits have on visitors themselves.

Several tools can be used to ensure that zoo visitors understand the importance of environmental enrichment for the animals, including educational signs, animal training and presentations, or having staff or volunteers draw the attention of visitors to the enrichment devices in an enclosure and clarify how the animals use them [1,21], especially in a walk-through enclosure [42] like the one in this study.

## 5. Conclusions

Our results suggest that visitors of the ring-tailed lemur enclosure were not affected by the appearance of the environmental enrichment items. Instead, visitor attitudes were affected to a greater extent by the activity and the behaviours that the animals displayed. Behavioural data on the activity of the ring-tailed lemurs, continuously recorded by the researchers during the experiment, as well as the visitors’ own reports of activity were shown to influence the visitor attitudes. Visitors found that the lemurs were well taken care of, scored the welfare of the lemurs high, and reported that both the enclosure and enrichment items were good-looking and appropriate for the species. We consider that our study supports the idea that zoo management decisions on environmental enrichment programmes should not only be based on the appearance of the enrichment devices, but on how they can stimulate certain species-specific behaviours in the animals. This would ensure better welfare for the animals in zoo settings, since they would benefit from good enrichment items regardless of their appearance, as well as enhance the experience of visitors.

## Figures and Tables

**Table 1:** Details of the ring-tailed lemurs involved in this study.

Lemur ID	Age	Sex	Offspring ID	Time Lived in this Zoo
1	15	Female	Lemur 2	4 years
2	9	Female	Lemurs 3 and 4	4 years
3	4	Female		From birth
4	2	Female		From birth
5	7	Male	Lemurs 3, 4 and 6	4 years
6	3	Male		From birth

**Table 2:** Details of the participants involved in this study. The two groups did not significantly differ in terms of age, gender, or the time they spent filling out the questionnaire.

	Naturalistic	Artificial	U/chi^2^
Mean age (SD)	35.41 years (12.1)	34.65 years (11.7)	0.784
Gender (male/female/unknown)	55/95/23	64/99/30	0.304
Mean time spent on the questionnaire (SD)	6.29 min (6.4)	5.71 min (2.7)	0.674

**Table 3:** Description of the environmental enrichment items used in this study. Several devices were built of each item using naturalistic and artificial-looking materials.

Item	Aim of the Device
Hanging baskets	To provide a new place for the lemurs to sit or swing on
Hammocks	To provide a new place to sit or swing on
Scent balls	To provide olfactory enrichment by filling burlap bags with aromatic stuffing inside (coffee, camel/sheep/horse hair, fruit tea, basil, pepper, and ginger)
Tube swings	To provide an elevated place to sit and swing and make foraging for food more challenging and time-consuming (the tubes were filled with food pellets or vegetable pieces)
Bottle feeders	To make foraging for food more challenging and time-consuming, since the lemurs had to manually turn the bottles until a food pellet or vegetable piece fell out

**Table 4:** Ethogram followed by the researchers, with the definition of each behaviour.

Behaviour	Definition
Active	Includes feeding/foraging and locomotion behaviour (walking, running, jumping and climbing)
Inactive	The individual is resting, sitting or sleeping
Social	The focal individual is playing with or grooming another individual
Interaction with enrichment	The individual is interacting with one or more enrichment devices
Inside	The individual is in the off-exhibit indoor enclosure and thus out of view
Other	Any other behaviour not included on the above list

**Table 5:** Relationship between visitor attitudes and the lemur behaviours as observed by the researchers. Only significant results are presented. *M* = mean; *B* = regression coefficient; *SE* = standard error; *Wald* = value of the Wald test for statistical significance.

Behaviour Reported by the Visitors	Likert-Scores	Statement	Statistical Details
Social behaviour	Lower	*The lemurs look stressed*	*M =* 2.02, *B* = -0.45, *SE* = 0.20, *Wald* = -2.26, *p* = 0.024
	Lower	*I think the enrichment disturbs the view of the enclosure*	*M* = 1.94, *B* = -0.43, *SE* = 0.21, *Wald* = -2.13, *p* = 0.033
	Higher	*I think the lemurs can behave as they would do in the wild*	*M* = 4.46, *B* = 0.50, *SE* = 0.19, *Wald* = 2.65, *p* = 0.008
	Higher	*I think this enclosure replicates the natural habitat of the ring-tailed lemurs well*	*M* = 4.57, *B* = 0.55, *SE* = 0.19, *Wald* = 2.86, *p* = 0.004
	Higher	*The lemurs look relaxed*	*M* = 5.98, *B* = 0.42, *SE* = 0.20, *Wald* = 2.14, *p* = 0.032
	Higher	*The lemurs look happy*	*M* = 5.76, *B* = 0.68, *SE* = 0.20, *Wald* = 3.40, *p* = 0.004
Feeding	Higher	*I think the enrichment in this enclosure is fit for the lemurs*	*M* = 5.73, *B* = 0.46, *SE* = 0.23, *Wald* = 1.98, *p* = 0.047
	Lower	*I think the enrichment disturbs the view of the enclosure*	*M* = 1.94, *B* = -0.55, *SE* = 0.23, *Wald* = -2.37, *p* = 0.018
Inactivity	Higher	*The lemurs look bored*	*M* = 2.63, *B* = 0.84, *SE* = 0.24, *Wald* = 3.50, *p*<0.001

**Table 6:** Relationship between visitor attitudes and the lemur behaviours as perceived by the visitors (binary: yes or no). Only significant results are presented. *M* = mean; *B* = regression coefficient; *SE* = standard error; *Wald* = value of the Wald test for statistical significance.

Behaviour Reported by the Visitors	Likert-Scores	Statement	Statistical Details
Social behaviour	Lower	*The lemurs look stressed*	*M =* 2.02, *B* = -0.45, *SE* = 0.20, *Wald* = -2.26, *p* = 0.024
	Lower	*I think the enrichment disturbs the view of the enclosure*	*M* = 1.94, *B* = -0.43, *SE* = 0.21, *Wald* = -2.13, *p* = 0.033
	Higher	*I think the lemurs can behave as they would do in the wild*	*M* = 4.46, *B* = 0.50, *SE* = 0.19, *Wald* = 2.65, *p* = 0.008
	Higher	*I think this enclosure replicates the natural habitat of the ring-tailed lemurs well*	*M* = 4.57, *B* = 0.55, *SE* = 0.19, *Wald* = 2.86, *p* = 0.004
	Higher	*The lemurs look relaxed*	*M* = 5.98, *B* = 0.42, *SE* = 0.20, *Wald* = 2.14, *p* = 0.032
	Higher	*The lemurs look happy*	*M* = 5.76, *B* = 0.68, *SE* = 0.20, *Wald* = 3.40, *p* = 0.004
Feeding	Higher	*I think the enrichment in this enclosure is fit for the lemurs*	*M* = 5.73, *B* = 0.46, *SE* = 0.23, *Wald* = 1.98, *p* = 0.047
	Lower	*I think the enrichment disturbs the view of the enclosure*	*M* = 1.94, *B* = -0.55, *SE* = 0.23, *Wald* = -2.37, *p* = 0.018
Inactivity	Higher	*The lemurs look bored*	*M* = 2.63, *B* = 0.84, *SE* = 0.24, *Wald* = 3.50, *p*<0.001

## Data Availability

The data presented in this study are available upon reasonable request.
